# 

**Published:** 1846-12

**Authors:** 


### TO READERS AND CORRESPONDENTS.

We have received communications from Drs. A. G. Henry, and Benton.

The following works have also been received:—

The Prescriber'sPharmacopœia, &c. By a Practising Physician. Revised and corrected by an American Physician. New York: Samuel S. and William Wood.1 846. pp. 144. 16mo. From the Publishers. For sale by Brautigam and Keen, Chicago.)

A Manual of Chemistry. By Richard D. Hoblyn, A.M. Oxon. &c. New York: Sam. S. & William Wood. 1846. pp. 336. (From the Publishers. For sale by Brautigam and Keen, Chicago.)

A Circular of the Committee of the National Convention, on Preliminary Education of Medical Students. From James Cowper, M.D., Chairman.

Annual Circular of the Medical Department of the University of Buffalo.

Medical Education in the United States: an Address delivered to the Students of the Philadelphia Association for Medical Instruction, at the Close of the Session of 1846. By Alfred Stille, M.D. Lecturer on Pathology and the Practice of Medicine. Phil. 1846. pp. 39. 8vo.

The following have been received in Exchange:

The Annalist, a Record of Practical Medicine. October 1, 1846. Vol. I. No. 1.

The Journal of Health and Monthly Miscellany, Boston. (In Exchange.)
The Medical Examiner. (In Exchange.)
The Bulletin of Medical Science. (In Exchange.)
The Western Journal of Medicine & Surgery. (In Exchange.)
The Buffalo Journal, and Medical Review. (In Exchange.)
Southern Medical & Surgical Journal. (In Exchange.)
The Western Lancet & Medical Library. (In Exchange.)
The St. Louis Medical and Surgical Journal. (In Exchange.)
The Medical News and Library. (In Exchange.)
The American Journal and Library of Dental Science. (In Exchange.)
The Boston Medical and Surgical Journal. (In Exchange.)
The Missouri Medical & Surgical Journal. (In Exchange.)
The New York Medical & Surgical Reporter. (In Exchange.)
The New York Journal of Medicine and the Collateral Sciences. (In Exchange.)

---

### CONTRIBUTORS TO THE ILLINOIS & INDIANA MED. & SURG. JOUR.

A. H. Howland, M. D , Ottawa, Ill.

A. G. Henry, M. D., Pekin, Ill.

Edward Lewis, M. D.. Jackson, Mich.

John McLean, M. D. Jackson, Mich.

Daniel Stahl, M. D.. Quincy, Ill.

Edward Mead, M. D. Geneva, Ill.

E. Fisher, Waynesville, Ohio.

H. S. Huber, M. D., Chicago, Ill.

Samuel G. Armor, M. D., Rockford, Ill.

Ira C. Bachus, M. D., Jackson, Mich.

J. G Cornell, M. D., Spring Arbor, Mich.

G. N. Fitch, M. D., Logansport, Ind.

John F. Henry, M. D., Burlington, Iowa.

E. Deming, M. D., Lafayette, Ind.

S. B. Thayer, Battle Creek, Mich.

---

Rock River Medical Society.—An adjourned meeting of this Society will be held at Dixon, Ill. on the third Tuesday of January. The Society is not limited in its boundaries. It is to be hoped, therefore, that every Physician who can will be present, to aid in advancing the interests of Medical Literature in the West.

S. G. Armor, M. D.
Secretary of the Society.

